# Robotic-assisted pancreatic surgery in the elderly patient: experiences from a high-volume centre

**DOI:** 10.1186/s12893-021-01395-w

**Published:** 2021-12-09

**Authors:** Karl H. Hillebrandt, Sebastian Knitter, Lea Timmermann, Matthäus Felsenstein, Christian Benzing, Moritz Schmelzle, Johann Pratschke, Thomas Malinka

**Affiliations:** 1 Department of Surgery, Charité – Universitätsmedizin Berlin, Freie Universität Berlin, Humboldt-Universität Zu Berlin, Berlin Institute of Health, Campus Virchow Klinikum I Campus Charité Mitte, Augustenburger Platz 1, 13353 Berlin, Germany; 2 Berlin Institute of Health at Charité – Universitätsmedizin Berlin, BIH Academy, Clinician Scientist Program, Charitéplatz 1, 10117 Berlin, Germany

**Keywords:** Robotic-assisted pancreatic surgery, Pancreatic surgery, Elderly patients

## Abstract

**Background:**

Robotic-assisted pancreatic surgery (RPS) has fundamentally developed over the past few years. For subgroups, e.g. elderly patients, applicability and safety of RPS still needs to be defined. Given prognosticated demographic developments, we aim to assess the role of RPS based on preoperative, operative and postoperative parameters.

**Methods:**

We included 129 patients undergoing RPS at our institution between 2017 and 2020. Eleven patients required conversion to open surgery and were excluded from further analysis. We divided patients into two groups; ≥ 70 years old (Group 1; n = 32) and < 70 years old (Group 2; n = 86) at time of resection.

**Results:**

Most preoperative characteristics were similar in both groups. However, number of patients with previous abdominal surgery was significantly higher in patients ≥ 70 years old (78% vs 37%, p < 0.0001). Operative characteristics did not significantly differ between both groups. Although patients ≥ 70 years old stayed significantly longer at ICU (1.8 vs 0.9 days; p = 0.037), length of hospital stay and postoperative morbidity were equivalent between the groups.

**Conclusion:**

RPS is safe and feasible in elderly patients and shows non-inferiority when compared with younger patients. However, prospectively collected data is needed to define the role of RPS in elderly patients accurately.

*Trial registration* Clinical Trial Register: Deutschen Register Klinischer Studien (DRKS; German Clinical Trials Register). Clinical Registration Number: DRKS00017229 (retrospectively registered, Date of Registration: 2019/07/19, Date of First Enrollment: 2017/10/18).

## Introduction

The field of minimally invasive surgery (MIS) has developed rapidly over the last decades, becoming state of the art for several procedures in visceral surgery [1, 2]. In the context of complex pancreatic procedures, MIS can already be seen as the new standard procedure for many indications showing similar or even better results compared to open surgery [3, 4]. For example, benign or low-grade malignant tumors of the pancreatic tail are mainly addressed by minimally invasive distal pancreatectomy [3]. Furthermore, Watson et al*.* demonstrated an improved oncological outcome in patients undergoing minimally invasive procedures for malignant indications [5].

One of the latest advances in MIS is robotic-assisted pancreatic surgery (RPS) [6]. While initial results seem promising, the exact role and indication for RPS, especially in the elderly patient, still needs a comprehensive assessment. In this regard, previously published experience on RPS remains scarce and is mainly based on a few retrospective studies [7, 8]. The first study on RPS was published in 2010 by Buchs et al*.* reporting on 15 robotic-assisted pancreaticoduodenectomies (RPD) in patients ≥ 70 years showing similar outcomes compared with younger patients [7]. A more recently published report by Liu et al. on RPD in elderly patients (≥ 75 years) showed increased postoperative morbidity and length of hospital stay [8].

Considering prognosticated upcoming demographic challenges, which forecast that individuals older than 85 years will have doubled by 2033, coupled with an increased incidence of hepato-pancreato-biliary (HPB) tumors demand further evidence on the role of RPS in this rapidly expanding cohort [9].

Altogether, we sought to contribute and extend recent experiences on the role of RPS in elderly patients aged ≥ 70 years in a high-volume centre with an established RPS program [10].

## Methods

### Data collection and definitions

Here we report the results of a single-center, prospective, post-marketing observational study (DRKS00017229, Date of Registration: 2019/07/19, Date of First Enrollment: 2017/10/18) with the objective of investigating clinical outcomes of robotic-assisted pancreatic surgery using the da Vinci Xi surgical system (Intuitive, Sunnyvale, CA, USA). All patients who underwent robotic-assisted pancreatic resection at the Department of Surgery, Campus Charité Mitte and Campus Virchow-Klinikum, Charité-Universitätsmedizin Berlin, Germany, between October 2017 and November 2020, were included. Written consent was obtained from all study participants. Baseline characteristics, intraoperative technical details including dissection devices, duration of surgery and console time as well as postoperative complications were prospectively recorded and analyzed.

All included patients gave informed consent to collection of their personal and medical data and its use for research purposes. All data were collected, stored and processed according to the General Data Protection Regulation and local data protection laws. The study was conducted in accord with the ethical standards of the Helsinki Declaration of 1975. The Charité institutional review board (IRB) approved of the study (CARE-Study (surgical assistance by robotic support; originally Chirurgische Assistenz durch Robotereinsatz, ethical approval code E/A4/084/17)).

Patients were divided into two groups; ≥ 70 years old (Group 1) and < 70 years old (Group 2). The cut-off was chosen according to previous studies published for a better comparison between the different cohorts [11]. Pre-, peri- and postoperative parameters were collected.

Experienced surgeons at the Charité Pancreatic Outpatient Centre advised the procedure specific indication for RPS and informed consent was collected from eligible patients. The da Vinci Xi surgical system (Intuitive, Sunnyvale, CA, USA) was applied for all procedures. All surgeons performing RPS were experienced in minimally invasive HPB procedures and had previously completed extensive robotic training [10].

Reasons for surgical conversion were sub-divided into three groups, (1) surgical or anatomical based (2) technical based and (3) patient- or anesthesia-related. Postoperative complications were classified using the Clavien-Dindo classification [12]. All procedure-related complications within 90 days (in-house or documented externally) were reviewed.

Major complications were defined as Clavien-Dindo grade ≥ 3. For grading of Postoperative Pancreatic Fistula (POPF), Postpancreatectomy Haemorrhage (PPH) and Delayed Gastric Emptying (DGE), the definitions of the “*International Study Group of Pancreatic Surgery*” were applied [13–15]. Surgical site infections (SSI) were classified according to the CDC definition [16]. Complications and death within 90 days determined postoperative morbidity and mortality.

### Statistical analysis

Quantitative and qualitative variables were expressed as medians (range) and frequencies. The Chi-square, Fisher's exact or Mann Whitney U test were used to compare variables between groups, as appropriate. Statistical analysis was performed using the SPSS software package, version 27, by IBM (Armonk, NY) and GraphPad PRISM (GraphPad Software, Inc., San Diego, CA). P values < 0.05 were considered to be statistically significant.

## Results

### Preoperative patients’ characteristics and indications

Between October 2017 and November 2020, 129 patients underwent RPS at the Charité – Universitätsmedizin Berlin, Campus Charité Mitte and Campus Virchow Klinikum in Berlin, Germany. Eleven patients required conversion to open surgery during RPS and were excluded from downstream analysis. We observed four (11%) surgical conversions in group 1 (≥ 70 years old) compared to seven (8%) conversions in group 2 (< 70 years old), showing no statistical difference (p = 0.499). Reasons for conversions are noted in Table 1.

**Table 1:** Indication for conversion during RPS

Characteristics	Group 1 ≥ 70 years old (n = 36)	Group 2 < 70 years old (n = 93)	*P*
Conversion, n (%)	4 (11)	7 (8)	0.499
Indication for conversion, n (%)			0.104
Surgical or anatomically	2 (50)	6 (86)	
Technical	0 (0)	1 (14)	
Patient- or anesthesia-related	2 (50)	0 (0)	

RPS, Robotic-assisted pancreatic surgery

Consequently, a total of 32 patients were assigned to group 1, while 86 patients were allocated to group 2. Table 2 indicates all preoperative patient characteristics and indications.

**Table 2:** Clinicopathological characteristics of 118 patients who underwent robotic-assisted pancreatic resection

Characteristics	Group 1 ≥ 70 years (n = 32)	Group 2 < 70 years (n = 86)	*P*
Sex, n (%)			0.142^a^
Female	19 (59)	38 (44)	
Male	13 (41)	48 (56)	
Median age at resection, years (range)	75.5 (70–87)	55.5 (22–69)	** < 0.0001**^b^
Median BMI, kg/m^2^ (range)	24.6 (18.0–35.4)	25.4 (18.5–41.9)	0.066^b^
BMI ≥ 30 kg/m^2^, n (%)	2 (6)	15 (17)	0.152^a^
Comorbidities, n (%)			
Cardiovascular disease	24 (75)	44 (51)	**0.020**^a^
Diabetes	6 (19)	9 (11)	0.231^a^
Pulmonary disease	2 (6)	13 (15)	0.350^a^
Renal insufficiency	1 (3)	1 (1)	0.471^a^
ASA physical status, n (%)			0.415^a^
I	0 (0)	4 (5)	
II	20 (63)	55 (64)	
III	12 (37)	27 (31)	
Malignant diagnosis, n (%)	21 (66)	47 (55)	0.284^a^
Diagnosis, n (%)			0.630^a^
Insulinoma	0 (0)	3 (4)	
Neuroendocrine tumor	1 (3)	5 (6)	
IPMN	7 (22)	13 (15)	
Pancreatitis	1 (3)	13 (15)	
MCN	1 (3)	2 (2)	
SCN	1 (3)	4 (5)	
SPN	0 (0)	2 (2)	
PDAC	12 (38)	26 (30)	
Periampullary carcinoma	7 (22)	12 (14)	
Intrapancreatic metastases	1 (3)	1 (1)	
Others	1 (3)	5 (6)	
Previous abdominal surgery, n (%)	25 (78)	32 (37)	** < 0.0001**^a^

Bold values are statistically significant

BMI, body mass index; ASA, American Society of Anesthesiologists; IPMN, Intraductal papillary mucinous neoplasm; MCN, Mucinous cystic neoplasm; SCN, Serous cystic neoplasm; SPN, Solid-pseudopapillary neoplasm; PDAC, Pancreatic ductal adenocarcinoma

^a^Chi-Square or Fisher`s Exact Test

^b^Mann Whitney Test

Both groups did not differ regarding gender distribution (59% females in group 1 and 44% females in group 2, p = 0.142) and the median BMI (24.6 kg/m^2^ in group 1; 25.4 kg/m^2^ in group 2 (p = 0.066)). ASA classification was similar in both groups (p = 0.415). Only the rate of cardiovascular comorbidities showed a significant difference between the two groups (p = 0.020). In patients aged ≥ 70 years old, 66% underwent RPS for malignant diagnoses compared to 55% in patients aged < 70 years old (p = 0.284). Patients in group 1 showed significantly more cases of previous abdominal surgery compared with patients in group 2 (78% vs. 37%, p < 0.0001).

### Procedures and intraoperative parameters

Detailed distribution of the procedures and intraoperative parameters can be found in Table 3. Procedures were evenly distributed between the groups. Hybrid procedures were performed in 18 patients in group 2 (21%) whereas in group 1 eight hybrid procedures were performed (25%, p = 0.635).

**Table 3:** Operative characteristics of 118 patients who underwent robotic-assisted pancreatic resection

Characteristics	Group 1 ≥ 70 years (n = 32)	Group 2 < 70 years (n = 86)	*P*
Primary procedure, n (%)			0.823^a^
Enucleation	0 (0)	2 (2)	
Distal pancreatectomy without splenectomy	1 (3)	3 (4)	
Distal pancreatectomy with splenectomy	13 (41)	36 (42)	
Appleby procedure	1 (3)	3 (4)	
Total pancreatectomy	0 (0)	3 (4)	
PPPD or Whipple procedure	17 (53)	39 (45)	
Concomitant procedure, n (%)	3 (9)	10 (12)	1^a^
Hybrid procedure, n (%)	8 (25)	18 (21)	0.635^a^
Median duration of operation (range), min	245.5 (62–532)	259 (56–535)	0.769^b^

PPPD, Pylorus-preserving pancreaticoduodenectomy

^a^Chi-Square or Fisher`s Exact Test

^b^Mann Whitney Test

The median operative time was 245.5 min in group 1 compared to 259 min in group 2 (p = 0.769).

### Postoperative course and complications

Postoperatively, 94% of the patients in group 1 were admitted to an intensive care unit (ICU) compared with 97% in group 2 (p = 0.612). While there was no significant difference for ICU admission after RPS, patients aged ≥ 70 years old had a significantly longer ICU stay than younger patients (1.8 vs 0.9 days; p = 0.037). However, for the length of the postoperative hospital stay no differences were observed. Readmission to ICU did not differ significantly between both groups (Group 1 16% vs Group 2 20%, p 0.607). The median hospital stay was equivalent between the groups with 11 (6–49) days in group 1 and 12 (4–91) days in group 2 (p = 0.707).

There was no difference in the overall postoperative morbidity when comparing older to younger patients (56% vs 69%; p = 0.210), and this remains unchanged when defining major postoperative morbidities (47% vs 57%; p = 0.327). The postoperative mortality rate was similar in both groups. While in group 1, only one patient (3%) died during a 90-day postoperative period, in group 2 four patients (5%) died. The distribution of the complications, according to the Clavien-Dindo classification, is documented in Table 4.

**Table 4:** Postoperative outcomes of 118 patients who underwent robotic-assisted pancreatic resection

Characteristics	≥ 70 years (n = 32)	< 70 years (n = 86)	*P*
Postoperative ICU stay, n (%)	30 (94)	83 (97)	0.612^a^
Median duration of ICU stay (range), days	1.8 (0.5–43.0)	0.9 (0.4–84.2)	**0.037**^b^
Median duration of hospital stay (range), days	11 (6–49)	12 (4–91)	0.707^a^
Postoperative morbidity, n (%)	18 (56)	59 (69)	0.210^a^
Major postoperative morbidity, n (%)	15 (47)	49 (57)	0.327^a^
Clavien Dindo classification, n (%)			0.958^a^
0	14 (44)	27 (31)	
I	1 (3)	5 (6)	
II	2 (6)	5 (6)	
IIIA	3 (9)	9 (11)	
IIIB	7 (22)	23 (27)	
IVA	3(9)	11 (13)	
IVB	1 (3)	2 (2)	
V	1 (3)	4 (5)	
Readmission to ICU, n (%)	5 (16)	17 (20)	0.607^a^
Readmission to hospital, n (%)	5 (16)	17 (20)	0.607^a^
Reoperation, n (%)	5 (16)	9 (11)	0.523^a^
POPF, n (%)			0.236^a^
No fistula	20 (63)	50 (58)	
A	1 (3)	9 (11)	
B	10 (31)	27 (31)	
C	1 (3)	0 (0)	
PPH, n (%)			0.358^a^
No haemorrhage	28 (88)	76 (88)	
A	0 (0)	2 (2)	
B	4 (12)	5 (6)	
C	0 (0)	3 (4)	
SSI, n (%)	3 (9)	3 (4)	0.342^a^
DGE, n (%)	4 (13)	7 (8)	0.487^a^
Anastomotic Leak of biliodigestive anastomosis, n (%)	2 (12)	2 (5)	0.571^a^
Patients with pulmonary complications, n (%)	9 (28)	14 (16)	0.149^a^
Pneumonia, n (%)	7 (22)	8 (9)	0.116^a^
Pleural effusion, n (%)	4 (13)	6 (7)	0.456^a^
Pulmonary embolism, n (%)	0 (0)	4 (5)	0.573^a^
Reintubation or tracheotomy, n (%)	2 (6)	5 (6)	1^a^
Pleural empyema, n (%)	0 (0)	2 (2)	1^a^
Patients with postoperative intervention, n (%)	13 (41)	46 (54)	0.236^a^
EGD for endogastric drainage, n (%)	6 (19)	21 (24)	0.515^a^
EGD for other reasons, n (%)	1 (3)	11 (13)	0.177^a^
ERCP, n (%)	1 (3)	5 (6)	1^a^
Coloscopy, n (%)	1 (3)	1 (1)	0.471^a^
New bile duct drain, n (%)	2 (6)	4 (5)	0.662^a^
New suction-irrigation drain, n (%)	8 (25)	27 (31)	0.499^a^
New other drain, n (%)	3 (9)	3 (4)	0.342^a^
Angiography for bleeding, n (%)	1 (3)	5 (6)	1^a^
Pre-emptive angiography, n (%)	2 (6)	2 (2)	0.297^a^
30-day mortality, n (%)	0 (0)	2 (2)	1^a^
90-day mortality, n (%)	1 (3)	4 (5)	1^a^

Bold value is statistically significant

ICU, intensive care unit; POPF, Postoperative pancreatic fistula; PPH, Post-pancreatectomy haemorrhage; SSI, Surgical site infection; DGE, Delayed gastric emptying

^a^Chi-Square or Fisher`s Exact Test

^b^Mann Whitney Test

Reoperations were necessary in five patients (16%) of group 1 and nine patients in group 2 (11%, p = 0.523), respectively. Postoperative interventions were performed on 41% of the patients in group 1 compared to 54% in group 2 (p = 0.236). The interventions that were necessary during the postoperative course can be found in Table 4. There was no difference for the individual interventions between the groups.

The clinically significant pancreatic fistulas (POPF B-C) occurred in 34% and 31%, in group 1 and 2, respectively (p = 0.758). PPH was observed in four patients in group 1 (12%) in which, however, no PPH C was documented. In group 2, PPH was 12% with three patients (3%) suffering from a PPH C event.

DGE and SSI showed no difference between the two groups (p = 0.487 and p = 0.342, respectively). Moreover, the number of insufficient biliodigestive anastomoses was also comparable in both groups. An occurrence of pulmonary complication was increased in group 1 (28%) compared to 16% in group 2 without reaching statistical significance (p = 0.149). A detailed list of pulmonary complications can be found in Table 4. Lastly, mortality at 30-days and 90-days was comparable between the groups (30-day mortality 0% vs 2%, p = 1; 90-day mortality 3% vs 5%, p = 1).

### Comparison of RDP and RPD

We further compared lengths of procedure and postoperative outcomes between RDP and RPD in both groups (see Table 5). No significant differences in median length of procedure could be found for RDP (w/o splenectomy) or RPD between both groups (p = 0.455 and p = 0.327), respectively. The median duration of the ICU stay after RDP was 1.4 days in group 1 compared to 1 day in group 2 (p = 0.513). For RPD, the median duration of the ICU stay 1.9 days in group 1 vs. 0.9 days in group (p = 0.087). Moreover, length of stay was equivalent for RDP and RPD between the two groups (RDP: p = 0.550; RPD: p = 0.858). Postoperative overall morbidity was 71% and 64% in group 1 and 2 after RDP (p = 0.748). After RPD a significantly lower postoperative morbidity could be observed in patients ≥ 70 years compared to patients < 70 years (47% vs. 77%, p = 0.028). However, postoperative major morbidity was comparable between the two groups after RDP (p = 0.905) and RPD (p = 0.294), respectively. After RPD, postoperative mortality was comparable after 30 (p = 1) and 90 days (p = 1). There was no postoperative mortality after RDP.

**Table 5:** Postoperative outcomes of 109 patients who underwent robotic-assisted DP or PPPD/Whipple

Characteristics	Distal pancreatectomy			PPPD or Whipple procedure		
	≥ 70 years (n = 14)	< 70 years (n = 39)	*P*	≥ 70 years (n = 17)	< 70 years (n = 39)	*P*
Median length of procedure (range), minutes	138 (62–353)	158 (62–330)	0.455^a^	300 (210–532)	308 (219–535)	0.327^a^
Median duration of ICU stay (range), days	1.4 (0.6–20.7)	1 (0.4–15.9)	0.513^a^	1.9 (0.5–43)	0.9 (0.6–84)	0.087^a^
Median duration of hospital stay (range), days	11 (6–44)	10 (5–52)	0.550^a^	12 (8–49)	15 (4–91)	0.858^b^
Postoperative morbidity, n (%)	10 (71)	25 (64)	0.748^a^	8 (47)	30 (77)	**0.028**^a^
Major postoperative morbidity, n (%)	8 (57)	23 (59)	0.905^a^	7 (41)	22 (56)	0.294^a^
Clavien Dindo classification, n (%)			0.539^a^			0.284^a^
0	4 (29)	14 (36)		9 (53)	9 (23)	
I	0 (0)	1 (3)		1 (6)	4 (10)	
II	2 (14)	1 (3)		0 (0)	4 (10)	
IIIA	1 (7)	6 (15)		2 (12)	2 (5)	
IIIB	4 (29)	12 (31)		3 (17)	10 (26)	
IVA	3 (21)	5 (13)		0 (0)	5 (13)	
IVB	0 (0)	0 (0)		1 (6)	2 (5)	
V	0 (0)	0 (0)		1 (6)	3 (8)	
30-day mortality, n (%)	0 (0)	0 (0)	–	0 (0)	2 (5)	1^a^
90-day mortality, n (%)	0 (0)	0 (0)	–	1 (6)	3 (8)	1^a^

Bold value is statistically significant

ICU, intensive care unit

^a^Chi-Square or Fisher`s Exact Test

^b^Mann Whitney Test

## Discussion

The aim of the present study was to assess the safety and feasibility of RPS in the elderly patient. Our results underline that RPS is safe and feasible, thus showing non-inferiority compared to younger patients.

RPS is one of the latest advances in minimally invasive surgery and receives a lot of attention [6]. Mainly for distal pancreatectomies published results of RPS seem promising, also with respect to the oncological outcomes [5]. Moreover, The International Study Group on Minimally Invasive Pancreatic Surgery documented that minimally invasive distal pancreatectomy may be superior to open surgery in selected cases [3]. The chosen technique (laparoscopic vs robotic) should be based on the experience and possibilities of the centre or surgeon [3]. Robotic-assisted pancreatoduodenectomy (RPD) still lacks sufficient prospective data to define its role and indication [3]. Although several publications have proven feasibility and safety of RPD compared to open surgery, prospective trials coupled with robust clinical data are still missing [17, 18].

Up to now, RPS in elderly patients remains a neglected topic with only few retrospective studies published to date [11]. We also lack sufficient evidence if RPS in elderly patients is associated with particular benefits compared to open pancreatic procedures.

Taking demographic changes into account, with likely more than 2 billion people aged above 60 projected in 2050, according to the WHO, more evidence is needed about RPS for elderly patients [19]. Another important aspect is the increased incidence of HPB malignancies in elderly patients [9, 20]. Regardless, radical surgical removal remains the only curative treatment available, although geriatric patients are at higher risk for developing postoperative complications [21]. Therefore, it seems meaningful to find beneficial surgical treatment options such as minimally invasive surgical approaches associated with reduced intraoperative blood loss, less postoperative complications, and shorter hospital stays [22].

We recently published our first experiences of RPS [10]. Our analysis demonstrates that RPS is safe and feasible for elderly patients compared to younger patients. No differences regarding important intraoperative and postoperative parameters could be observed in both groups, making RPS a suitable approach in the elderly patient.

Our results correspond with Buchs et al. although they specifically focused solely on RPD in elderly patients [7]. However, a more recently published study by Liu et al. showed different results [8]. Their data on RPD in patients aged 75 or older documented higher postoperative morbidity and longer length of hospital stay. Furthermore, their cohort included elderly patients showing higher ASA scores, which possibly explains their observations in the postoperative course of elderly patients. In our cohort, the ASA scores and the comorbidities showed no differences between the elder and younger patients, a circumstance that may underscore the need for rigorous indications for RPS in elderly patients in order to achieve similar positive postoperative outcomes compared to younger patients.

Our cohort's only relevant preoperative difference was the number of previous abdominal surgeries, which were significantly higher in group 1, which likely relates to the accumulation of abdominal surgeries with age. However, this factor did not result in changes of the conversion rate, operation time or postoperative outcome; seemingly counterintuitive, as one could have expected more strenuous adhesiolysis with concurring negative side effects in those patients.

However, our findings are supported by similar results from laparoscopic liver surgery within our institution, where previous abdominal surgeries did not influence operative time and postoperative outcome [23].

The length of hospital stay in our groups did not differ and is comparable to other published cohorts on RPS [24]. However, we did observe a significant difference between elderly patients and younger patients regarding the length of their ICU stay, which was higher in group 1. This could be related to the general health status and careful handling of these patients on the ICU to prevent readmission. Regarding surgical and non-surgical patients, it has generally been shown that older patients have increased hospital mortality aligned with a prolonged time on ICU [25].

However, in our cohort, the length of ICU stay did not influence the postoperative length of hospital stay, morbidity or mortality in patients aged ≥ 70 years, most likely as a result of rigorously selected patients undergoing RPS at our institution.

Overall morbidity and mortality did not differ significantly between the two groups. At first sight, overall morbidity seems to be high in our cohort but is comparable to other newly established RPS programs [24, 26, 27]. First, this is known to be associated with the initial learning curve in RPS, well documented by several previous studies [27, 28]. Second, this could also be related to the cohort's heterogeneity regarding the main and concomitant procedures. Lastly, the rate of major morbidity (Clavien-Dindo ≥ 3) seems high compared to other published cohorts. An incidence may be related to our centre-specific approach to internalize drainages early by using an endoscopic approach [29]. After RPD, we observed a significantly lower postoperative overall morbidity in patients aged ≥ 70 years than in patients aged < 70 years. This finding is probably related to the highly selected patient cohort in group 1. However, postoperative major morbidity for RPD did not significantly differ between the two groups.

Nevertheless, this study has some limitations. First, due to the observational study character, the small group size and the heterogeneous group composition, our conclusions remain limited. Furthermore, our results and findings are from a single centre and may therefore not be generally applicable. Another critical point might be our selection bias for patients who are suitable for RPS, especially in the patients ≥ 70 years. This results in a highly pre-selected cohort for RPS. Moreover, sufficient data on postoperative quality of life and long-term oncological outcomes are still missing. Thus, prospective studies are urgently needed to investigate feasibility, safety and outcomes of RPS in the elderly patient.

## Conclusion

Our results demonstrate that RPS is feasible and safe in the elderly patient and does not increase postoperative morbidity, mortality or length of hospital stay. However, further prospective studies are needed to define the role of RPS in elderly patients to accommodate the upcoming demographic change.

## Data Availability

The data that support the findings of this study are available on request from the corresponding author. The data are not publicly available due to privacy or ethical restrictions.

## Abbreviations

ASA: American Society of Anesthesiologists
CDC: Centers for Disease Control and Prevention
DGE: Delayed Gastric Emptying
HPB: Hepato-Pancreato-Biliary
ICU: Intensive Care Unit
IRB: Institutional Review Board
MIS: Minimally Invasive Surgery
POPF: Postoperative Pancreatic Fistula
PPH: Postpancreatectomy Haemorrhage
RDP: Robotic-assisted Distal Pancreatectomy
RPD: Robotic-assisted Pancreaticoduodenectomy
RPS: Robotic-assisted Pancreatic Surgery
SSI: Surgical site infections
WHO: World Health Organization
